# Genomic Epidemiology of Rift Valley Fever Virus Involved in the 2018 and 2022 Outbreaks in Livestock in Rwanda

**DOI:** 10.3390/v16071148

**Published:** 2024-07-17

**Authors:** Isidore Nsengimana, John Juma, Kristina Roesel, Methode N. Gasana, Fabrice Ndayisenga, Claude M. Muvunyi, Emmanuel Hakizimana, Jean N. Hakizimana, Gillian Eastwood, Augustino A. Chengula, Bernard Bett, Christopher J. Kasanga, Samuel O. Oyola

**Affiliations:** 1 Department of Veterinary Microbiology, Parasitology and Biotechnology, Sokoine University of Agriculture, Morogoro P.O. Box 3000, Tanzania; 2 SACIDS Africa Centre of Excellence for Infectious Diseases, SACIDS Foundation for One Health, Sokoine University of Agriculture, Morogoro P.O. Box 3297, Tanzania; 3 Rwanda Inspectorate, Competition and Consumer Protection Authority, Kigali P.O. Box 375, Rwanda; 4 Department of Entomology, and Center for Emerging Zoonotic & Arthropod-Borne Pathogens (CeZAP), Virginia Polytechnic Institute and State University, Blacksburg, VA 24061, USA; 5 International Livestock Research Institute (ILRI), Nairobi P.O. Box 30709, Kenya; 6 Department of Animal Resource Research and Technology Transfer, Rwanda Agriculture and Animal Resources Development Board (RAB), Huye P.O. Box 5016, Rwanda; 7 Rwanda Biomedical Center (RBC), Kigali P.O. Box 7162, Rwanda

**Keywords:** Rwanda, RVFV, outbreak, genome, sequence, phylogeny

## Abstract

Rift Valley fever (RVF), a mosquito-borne transboundary zoonosis, was first confirmed in Rwanda’s livestock in 2012 and since then sporadic cases have been reported almost every year. In 2018, the country experienced its first large outbreak, which was followed by a second one in 2022. To determine the circulating virus lineages and their ancestral origin, two genome sequences from the 2018 outbreak, and thirty-six, forty-one, and thirty-eight sequences of small (S), medium (M), and large (L) genome segments, respectively, from the 2022 outbreak were generated. All of the samples from the 2022 outbreak were collected from slaughterhouses. Both maximum likelihood and Bayesian-based phylogenetic analyses were performed. The findings showed that RVF viruses belonging to a single lineage, C, were circulating during the two outbreaks, and shared a recent common ancestor with RVF viruses isolated in Uganda between 2016 and 2019, and were also linked to the 2006/2007 largest East Africa RVF outbreak reported in Kenya, Tanzania, and Somalia. Alongside the wild-type viruses, genetic evidence of the RVFV Clone 13 vaccine strain was found in slaughterhouse animals, demonstrating a possible occupational risk of exposure with unknown outcome for people working in meat-related industry. These results provide additional evidence of the ongoing wide spread of RVFV lineage C in Africa and emphasize the need for an effective national and international One Health-based collaborative approach in responding to RVF emergencies.

## 1. Introduction

Rift Valley fever (RVF) is a zoonotic mosquito-borne viral hemorrhagic fever caused by RVF virus (RVFV), a member of the *Phlebovirus* genus and *Phenuiviridae* family [1]. This transboundary arboviral disease of great One Health and economic importance came to the attention of East African British colony officials in early 1900s as an obscure disease causing heavy mortality in young lambs born from exotic sheep breeds [2]. It was described for the first time in 1930 in a Merino sheep farm in the Rift Valley region of Kenya [3], while in the same year RVF-like cases were also recorded by the Tanzanian Ministry of Livestock and Fisheries [4]. Since that time, RVFV has emerged with great potential to transmit across borders and currently is endemic in many African countries and the Arabian Peninsula [5]. This phlebovirus is readily transmissible to animals and humans with the capacity to cause severe disease. In domesticated ruminants such as sheep, goats, and cattle, the disease is characterized by fever, hemorrhage, abortion storm, and high mortality in young animals [3,5,6]. In humans, although most cases are asymptomatic or self-limiting febrile illnesses, a small proportion (1–2%) of cases results in severe complications such as hepatitis, encephalitis, blindness, hemorrhagic syndrome, and death [7,8]. The case fatality rate is estimated to be 0.5–2% [9], although it is higher among hospitalized patients [10,11,12].

RVFV has caused large and devastating epizootics and epidemics in Africa and the Arabian Peninsula [13]. Since 2000, outbreaks have been reported in countries such as Saudi Arabia in 2000–2001 [10], Kenya, Tanzania, and Somalia in 2006–2007 [10], Sudan in 2007 and 2010 [14], Madagascar in 2008 [15], South Africa 2008, 2009–2011 [16], and 2018 [17], Namibia in 2011 [18], Mauritania in 2010 [19], Niger in 2016, Rwanda in 2018 [20] and in 2022 [21], and in Uganda, where sporadic-only outbreaks were recorded from 2016 to 2022 [22,23,24]. The outbreaks are often preceded by periods of above-normal rainfall with flooding, which lead to increased mosquito populations [25,26]. RVFV is primarily transmitted by infected mosquitoes to animals, which then amplify the virus, while other blood-sucking insects can transmit the virus mechanically [27]. A wide range of mosquito species have been suggested as potential vectors for RVFV transmission [13]. Members of *Aedes* and *Culex* mosquito genera have been identified as the major primary vectors [28]. Human infection occurs through direct contact with infected animals or their tissues, or by infected mosquito bites [29]. No human-to-human transmission has been reported [30].

RVFV is known to cause recurrent outbreaks with variable inter-epidemic periods. In Tanzania, the inter-epidemic period was found to be between 3 and 17 years (7.9 years average) for 10 well-described outbreak waves [4], while in Kenya, the intervals were shorter, between 1 and 7 years (3.9 years average) for 11 national epizootics analyzed [31]. The virus is believed to be maintained between epidemics through desiccant-resistant transovarially infected eggs of *Aedes* mosquitoes [32] or permanent silent circulation between the mosquito vectors and vertebrate hosts [11,33,34]. Recent reports indicate that the latter mechanism is the more predominant, and the long dormancy in mosquito eggs initially suggested may not be necessary in regions with the permanent presence of mosquitoes [35,36,37]. A wide range of wildlife species, including bats and rodents, have been found to be seropositive against RVFV [38,39,40], but the natural vertebrate reservoir is not yet known [38].

Like other bunyaviruses, RVFV is an enveloped, single-stranded, negative-sense RNA virus with a tri-segmented genome of about 11.6 kilobase pairs (kb) [1]. The large (L) RNA segment (~6.4 kb) encodes the RNA-dependent RNA polymerase (L) protein. The medium (M) segment (~3.9 kb) encodes in its single open reading frame the 14 kDa non-structural protein (NSm), a 78 kDa Gn/NSm fusion protein, and two major viral envelop glycoproteins, Gn and Gc, used for viral attachment and entry. The S segment (~1.7 kb), which is ambisense, encodes the nucleocapsid (N) protein in the genomic sense and the non-structural protein (NSs) in the antigenomic sense. The NSs protein is a major virulence factor interfering with the host cell transcription and interferon response [41]. The RVFV genome is highly conserved, and the virus has been so far known as a single serotype [42]. Genome variability was approximated to be 4–5% among geographically and temporally diverse RVF strains [43]. Although segment reassortment has been reported in RVF viruses [44,45], most genome variations have occurred as random single nucleotide polymorphisms (SNPs) with no well-defined variable sites [41].

The potential of RVFV to spread across geographical borders has raised global concern. The disease, initially confined to the sub-Saharan Africa, moved northwards to Egypt in 1977 [46], and was first isolated in Madagascar in the Indian Ocean in 1979 [15]. It invaded Mauritania in West Africa in 1987 [47], and appeared in the Asian continent in Saudi Arabia and Yemen by 2000 [10]. The mechanisms of RVF virus movement and spread remained less understood until the discovery and application of sequencing and pathogen genomics [42,48]. Advances in next generation sequencing (NGS) coupled with increased computational capability for molecular analysis have enabled precise characterization of the circulating pathogen strains, their origin, and transmission dynamics [49,50,51]. However, several challenges, such as high cost and the need for high performance computing equipment and human resource capacity, still limit the wide use of such genome sequencing technology, making pathogen characterization incomplete in many resource-limited settings [42]. Molecular epidemiological studies show that RVF outbreaks can occur in novel geographical settings following a new introduction event [15,18,22,24,46,52,53] or re-activation of resident single- or multi-virus lineage strain(s) [44,54]. RVFV strains have been so far classified into 15 lineages, named from A–O [48], and these genotypes have been characterized by widespread dispersal events throughout Africa [43].

In Rwanda, RVFV was first confirmed in the Bugesera district in 2012 as the cause of abortion storms observed in cattle about two years earlier. Confirmation by both enzyme -linked immunosorbent assay (ELISA) and reverse-transcription polymerase chain reaction (RT-PCR) tests led to the immediate start of a RVF vaccination campaign in the country [55]. A serological study conducted on 595 cattle samples collected in 6 districts between December 2012 and March 2013 showed an overall seroprevalence of 16.8% [56]. Since that time, RVFV circulation in Rwanda has resulted in sporadic outbreaks reported to the World Organization for Animal Health (WOAH) almost every year [57], until the occurrence of a first large outbreak in 2018. This outbreak, which started during the long rainy season (March–May) and was confirmed in May 2018, started in the Eastern province of Rwanda and spread to 12 of the 30 districts of the country [20]. After an inter-epidemic period of four years, a further large outbreak occurred in Rwanda in 2022, while the country was simultaneously recovering from the consequences of COVID-19 pandemic. This second outbreak was confirmed in March 2022 during the rainy season, similar to the previous outbreak [21]. Unlike in 2018, the 2022 outbreak expanded to all 30 districts of the country (unpublished), and cases were detected up until December 2022. Despite the evident risks of RVF in Rwanda, little information is available on the underlying epidemiology of the disease. The origin and genetic characteristics of the viruses involved in both outbreaks have remained unknown. The objective of this molecular epidemiological study was to conduct a genomic analysis of RVFV detected in livestock samples and determine the genotypes and ancestral origin of RVF viruses that circulated in Rwanda during the 2018 and 2022 outbreaks.

## 2. Materials and Methods

### 2.1. Study Area

The Republic of Rwanda is a land-locked country with an area of 26,338 km^2^, situated between East and Central Africa. The country shares borders with the Democratic Republic of the Congo (DRC) in the west, Uganda to the north, Tanzania to the east and Burundi in the south. Rwanda is divided into five administrative provinces, namely East, South, West, North, and the City of Kigali. The climate is bimodal; a short rainy season spans from September to December, a long rainy season from March to May, a short dry season from January to February, and a long dry season from June to August. Annual temperature varies according to elevation, with an annual average of 19 °C and annual rainfall ranging between 900 and 1600 mm [58]. Agriculture is an important activity, engaging 80.1% of all Rwanda’s households [59]. Due to its economic, food security, and socio-cultural importance, cattle farming predominates in animal agriculture, and 53% of all households rear cattle. The country owns approximately 1.6 million cattle [59], of which more than a quarter (~430,000) are dairy cows that have been given to poor families through a Government program named “One Cow Per Family” since its initiation in 2006 [60].

### 2.2. Sample Collection

**Figure 1:**
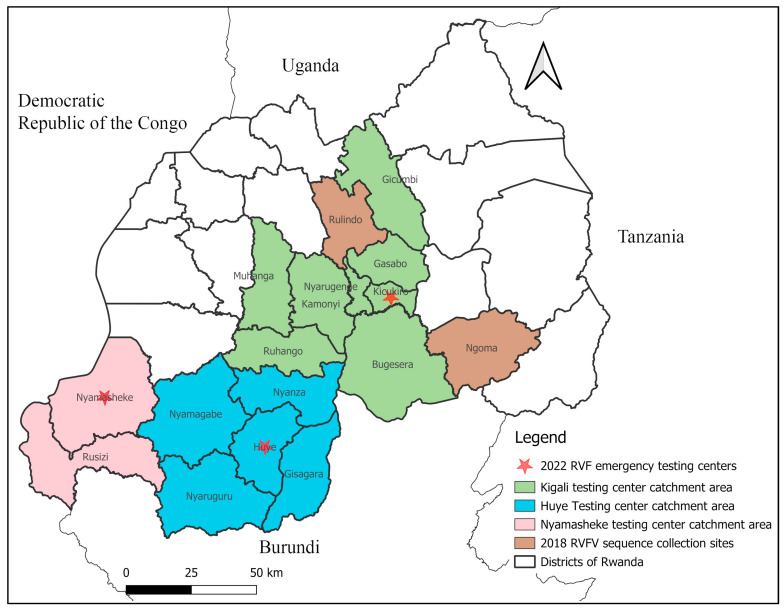
Administrative map of Rwanda, showing in red stars the location of RVF emergency testing centers where slaughterhouse samples used in this study were screened during the 2022 Rwanda RVF outbreak. The green, blue, and pink colors show the Kigali, Huye and Nyamasheke RVF testing center catchment areas, respectively. The dark brown color indicates collection sites for RVFV successfully sequenced for the 2018 outbreak. The map was drawn using QGIS version 3.24.1, freely available at https://www.qgis.org/en/site/, accessed on 25 May 2024.

The livestock samples used in this study were obtained from the Rwanda Agriculture and Animal Resource Development Board (RAB), Central Veterinary Laboratory, located in Rubirizi, Kigali. A total of 37 archived and unscreened samples (9 sera and 28 whole blood samples) from the 2018 RVF outbreak were collected between 14 April and 17 July 2018 from six districts of the Eastern province and the Rulindo district of the Northern province. In addition, 157 archived RVFV-positive samples (as detected by RT-qPCR) were available from the 2022 RVF outbreak, and originated from three (Kigali, Huye, and Nyamasheke) of the six RVF emergency testing centers established by the Government of Rwanda as part of the response to the 2022 RVF outbreak. These centers were charged to systematically perform pre-slaughter RVFV screening by RT-qPCR of all animals arriving at slaughterhouses (both large and small) for meat production. The purpose of this screening was to prevent human infections that would be caused by handling infected animals and derived meat and other tissues. Each center processed the samples aggregated from different slaughterhouses operating in their assigned catchment area (Figure 1). At each slaughterhouse, animal blood samples were drawn from the jugular vein in 4 mL ethylenediaminetetraacetic acid (EDTA)-coated vacutainer tubes by trained field veterinary technicians wearing appropriate personal protective equipment (PPE). After collection, blood samples were placed in cooler boxes and transported to the testing centers.

### 2.3. Molecular Screening of Samples during the 2022 RVF Outbreak

Blood samples were centrifuged at 4000 rpm for 3 min in order to separate out the plasma. Samples were tested in pools, where a pool comprised 10 samples aggregated in one tube by mixing 200 μL of plasma from each sample. For every pool that tested positive, each of the 10 samples within that pool was tested individually to distinguish positive from negative samples. Total RNA was extracted from samples using the automated Maxwell nucleic acid extraction system (Promega Corporation, Madison, WI, USA) according to the manufacturer’s instructions. RVFV RNA was detected on a Quant Studio 5 Thermo-cycler (Applied Biosciences, Foster City, CA, USA) by RT-qPCR using a commercial kit, RealStar^®^ Rift Valley Fever Virus RT-qPCR Kit (Altona Diagnostics, Hamburg, Germany) in accordance with manufacturer instructions. Individual positive samples, archived with required identification information, were selected for this study and shipped in dry ice to the International Livestock Research Institute (ILRI), Nairobi, Kenya for RVFV genome sequencing.

### 2.4. Pre-Sequencing RT-qPCR

Total RNA was extracted from 200 μL of each animal sample (serum, plasma, or whole blood) using a QIAamp viral RNA extraction mini kit (Qiagen, Hilden, Germany) and automated nucleic acid extraction system (TANBead Inc., Taoyuan, Taiwan) according to the manufacturer’s instructions. The purified RNA was collected in a final volume of 50 µL of elution buffer. RVFV RNA was detected using a TaqMan 1-step RT-qPCR master mix (Applied Biosystems, Waltham, MA, USA) and the primer-probe set as described by Bird et al. [61]. This primer-probe set amplified a highly conserved region of the virus L-segment. The RT-qPCR was run on the Quant Studio 5 PCR thermocycler under the following conditions: 50 °C for 10 min, 95 °C for 2 min, 95 °C for 3 s and 60 °C for 30 s. Samples with a cycle threshold (CT) value less than 30 were selected for sequencing, as this subset criterion had previously shown a higher probability of yielding a complete genome sequence [44,51].

### 2.5. Sequencing and Consensus Sequence Generation

In order to generate the genome sequences, the Multiplex PCR Amplicon sequencing approach was used as described by Juma et al. [51]. Briefly, a LunaScript RT Supermix Kit (New England Biolabs, Hitchin, UK) was used for reverse transcription of RNA into cDNA according to the manufacturer’s instructions. The cDNA was then amplified in two individual amplicon multiplex PCR reactions using 76 primers (38 odd-numbered primers in reaction one, and 38 even-numbered primers in reaction two) deployed to overlap the three segments of the virus genome in the order of 6, 12, and 20 primer pairs covering the S, M, and L segments, respectively. Initial cDNA denaturation was performed at 95 °C for 30 s, with 35 subsequent amplification cycles of 95 °C for 15 s (denaturation) and 63 °C for 5 min (annealing/elongation), before holding at 4 °C. Libraries were prepared using a Next Ultra II DNA Library Prep Kit (New England Biolabs, Hitchin, UK) and loaded onto the Illumina (NextSeq 550) sequencing platform according to the manufacturer’s instructions. The raw sequence data generated were demultiplexed, cleaned to remove reads of low quality, and trimmed to remove adapter sequences. High-quality reads were analyzed using a rvfv-amplicon-seq nextflow pipeline [62]. Briefly, raw sequence reads were assessed on quality using FASTQC [63], with trimming of low-quality genomic data with FASTP [64]. Pre-processed reads were aligned to the RVFV reference (ZH548 strain) using bwa-mem [65]. Amplicon primer sequences were trimmed using iVAR [66]. Variant calling was performed to generate consensus sequences for positions with over 10x coverage and base quality scores of 20 and above. Consensus sequences with at least 90% coverage were selected for downstream analyses, except for four sequences of the M segment (P120, P323, P68, and P118), considered with a genome coverage between 87.8 and 89.8% to have a good representation of this sequence cluster in our analysis.

### 2.6. Maximum Likelihood (ML) and Molecular Clock Phylogenetic Analysis

Reference sequences for RVFV segments L, M, and S were selected from GeneBank [https://www.ncbi.nlm.nih.gov/] (Supplementary Materials, Tables S1–S3 using Basic Local Alignment Search Tool for nucleotides (BLASTn)). MEGA XI software [67] was used for the initial analyses of each genome segment sequence dataset. Sequences were aligned using ClustalW [67] and the pairwise nucleotide difference was computed via pairwise distance. The best-fitting nucleotide substitution model for each genome segment dataset was selected based on the lowest Bayesian information criterion (BIC) value. The maximum likelihood (ML) phylogenetic tree for each segment was reconstructed with bootstrap support provided by 1000 iterations. A further category of analyses used the Bayesian analysis software packages, namely BEAST v1.10.4, BEAUti v1.10.4, Tracer.v1.7.2 [68], and TempEst v1.5.3 [69]. The latter was used to check the temporal signal in the data and their fitness for molecular clock analysis. After multiple runs of BEAST—Tracer, Bayesian analyses were completed with random starting trees together with the Tamura-Nei (TN93) nucleotide substitution model, a strict molecular clock, and coalescent constant size [70] tree priors, as well as the Markov chain Monte Carlo (MCMC) chain length of 2 × 10^7^ with a 20% burn-in allowing an effective sample size of at least 200. Phylogenetic trees were summarized with Tree Annotator v1.10.4 [68] and visualized with FigTree.v1.4.4 [71]. To characterize the lineages from the consensus RVFV genomes, we utilized both the command line (https://github.com/ajodeh-juma/rvfvtyping, accessed on 12 July 2023) and online (https://www.genomedetective.com/app/typingtool/rvfv/, accessed on 10 July 2023) versions of the RVFV lineage assignment tool [72]. RVFV genome sequences obtained in this study were deposited into GenBank and given accession numbers (PP746304–PP746414). Further details of sequences are shown in the supplementary material under Table S4.

## 3. Results

### 3.1. RVFV Genome Sequencing

From a total of 37 livestock samples processed for the 2018 Rwanda RVF outbreak, two geographically distant isolate sequences with 94–96% genome coverage were obtained, one from Ngoma district, Eastern province, and the other from Rulindo district, Northern province (Table S5). Further, a total of 38, 41, and 36 sequences (with 87.8–98% genome coverage) for L, M, and S segments, respectively, were recovered from 157 slaughterhouse samples processed for the 2022 Rwanda RVF outbreak (Table S6).

### 3.2. Phylogeny and Lineage Assignment

**Figure 2:**
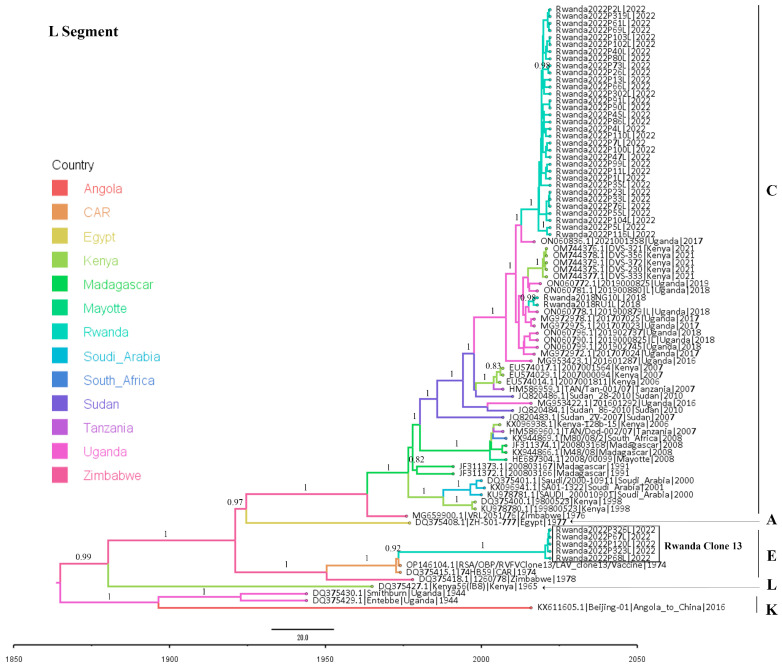
Bayesian molecular clock analysis of RVFV strains based on L segment. The time-scaled maximum clade credibility tree is depicted with branches colored according to country of origin of the viruses analyzed. The phylogenetic tree tips are labeled as accession, name, country, and year of collection of the specimens. RVFV Clone 13 sequences isolated in Rwanda are shown in the box. The letters indicate RVFV lineages. Posterior support values (HPD) are shown above each node (i.e., 1 = 100% support).

**Figure 3:**
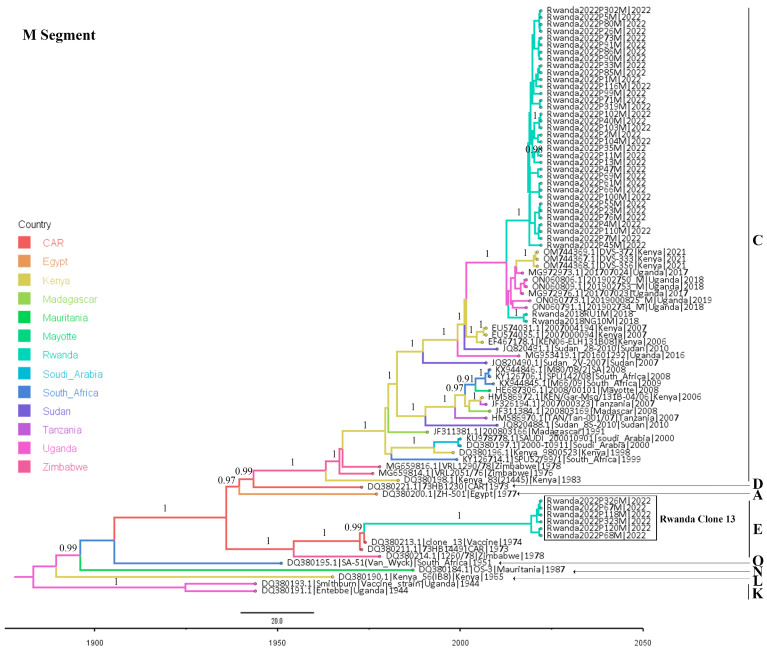
Bayesian molecular clock analysis of RVFV strains based on M segment. The time-scaled maximum clade credibility tree is shown with branches colored according to country of origin of the viruses analyzed. The phylogenetic tree tips are labeled as accession, name, country, and year of collection of the specimens. RVFV Clone 13 sequences isolated in Rwanda are shown in the box. The letters indicate RVFV lineages. Posterior support values (HPD) are shown above each node (i.e., 1 = 100% support).

**Figure 4:**
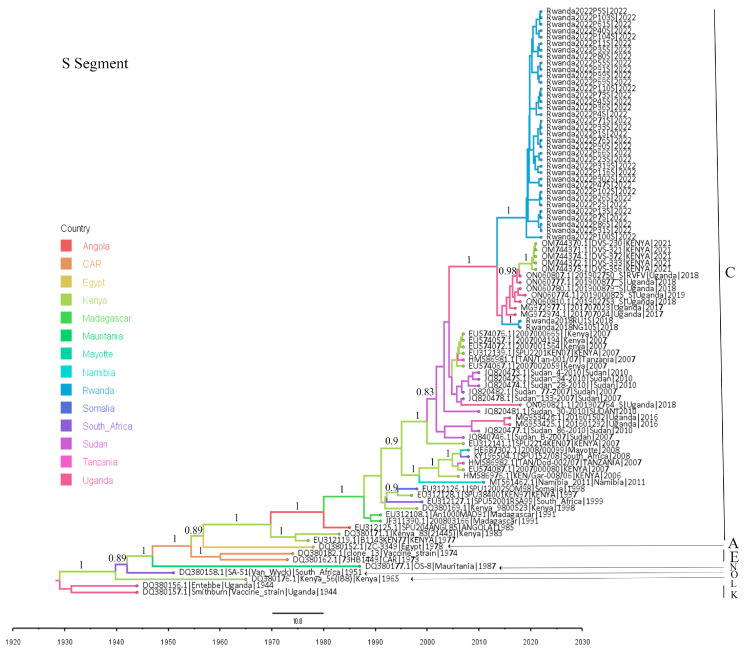
Bayesian molecular clock analysis of RVFV strains based on S segment. The time-scaled maximum clade credibility tree is presented with branches colored according to country of origin of the viruses analyzed. The phylogenetic tree tips are labeled as accession, name, country, and year of collection of the specimens. RVFV Clone 13 sequences isolated in Rwanda are shown in the box. The letters indicate RVFV lineages. Posterior support values (HPD) are shown above each node (i.e., 1 = 100% support).

Both ML- and Bayesian-based phylogenetic analyses as well as genetic lineage assignment were completed. The three segment-based phylogenetic trees obtained by both analysis methods exhibited congruent topologies. The Bayesian molecular clock phylogenetic trees for the three RVFV genome segments are presented in Figure 2, Figure 3 and Figure 4. The ML phylogenetic trees are shown in the Supplementary Material (Figures S1–S3), and the lineage assignment output is shown in Table S7.

These analyses indicated that the Rwandan RVFV sequences clustered into three clades belonging to the circulating lineage C and the vaccine virus lineage E. The two sequences from the Rwanda 2018 outbreak, despite their wide geographical distance, were very similar to each other (pairwise nucleotide identity difference between 0 and 0.05% across the three virus segments) and clustered in lineage C, sharing their most recent common ancestor with the viruses that were detected in Uganda between 2016 and 2019.

The RVFV sequences obtained for the 2022 outbreak sorted into two groups, namely a small group made up of six sequences (and five retained for the L segment) which were most similar to the RVFV Clone 13 vaccine strain [73] used in Rwanda [74] during the 2022 outbreak (vaccine-derived viruses), and a large group comprising 38, 41, and 36 wild-type virus sequences for the L, M, and S segments, respectively. The vaccine-derived viruses, which were also closely related to each other (100% pairwise nucleotide identity across the L and M segments), exhibited a very high identity with the RVFV Clone 13 strain, showing only a nucleotide identity difference between 0 and 0.03% across both the L and M segments. Both maximum likelihood and Bayesian-based phylogenetic trees reconstructed for the L and M segments indicated that these vaccine-derived viruses’ most recent ancestor was RVFV Clone-13, the vaccine strain classified in the lineage E. The S segments of these six vaccine-derived viruses were poorly sequenced, with genome coverage between 49.1 and 59.5%, and were thus not considered for the S segment analysis. However, analysis performed using short sequences of the S segments recovered from these viruses showed a similar tree topology.

Like the 2018 Rwanda RVFV outbreak sequences, the large group of 2022 outbreak wild-type viruses comprised highly identical viruses despite their large number and geographical distances. This monophyletic relationship was supported by ML bootstrap values of 99–100% across viral segments. Their pairwise comparison showed a nucleotide identity difference of 0–0.12% in L segments, 0–0.17% in the M segment and 0–0.44% in the S segment. Similar to their counterparts from the 2018 Rwanda RVF outbreak, the analyses showed that the 2022 Rwanda outbreak wild-type viruses were also grouped in lineage C, sharing a most recent common ancestor with a large virus cluster comprising specimens isolated in Uganda in 2016–2019, Rwanda in 2018, and Kenya in 2021.

Comparing the viruses of the two outbreaks, a very close phylogenetic relatedness was observed between the Rwanda 2022 outbreak wild-type viruses and the Rwanda 2018 isolates, with a maximum average nucleotide identity difference of 0.61% across the three segments (0.22–0.31% in the L segments; 0.34–0.45% in the M segments; and 0.46–0.90% in the S segments. There was no evidence of segment reassortment found in the sequence data. The observed congruency of phylogenetic tree topologies, even with the L and M segment trees containing live-attenuated vaccine-recovered sequences, indicated the absence of segment reassortment in the analyzed sequence data.

### 3.3. Molecular Evolutionary Rates and Time to the Most Recent Common Ancestor (MRCA)

The Bayesian molecular clock analysis of a data set comprising Rwandan RVFV sequences generated evolutionary rates with ranges comparable to those reported previously [15,44]. The mean evolutionary rate and the 95% high posterior density (HPD) interval for each segment were as follows: S segment, 5.1 × 10^−4^ (4.2 × 10^−4^ to 6.2 × 10^−4^); M segment, 2.7 × 10^−4^ (2.2 × 10^−4^ to 3.2 × 10^−4^); and L segment, 1.7 × 10^−4^ (1.4 × 10^−4^ to 2.1 × 10^−4^) nucleotide substitutions/site/year. The time-scaled clade credibility phylogenetic trees reconstructed indicate that the most recent common ancestor for both the Rwanda 2018 and 2022 RVF outbreak viruses existed around 2010 (in the L segment), 2012 (in the M segment), and in 2013 (in the S segment). It could also be noted that this common ancestry date shifted to 2007 when Uganda RVFV sequences were also considered. This year of 2007 coincided with the large 2006/2007 Eastern Africa RVF outbreak reported in Kenya, Tanzania, and Somalia [54].

## 4. Discussion

Rwanda confirmed its first cases of RVF in 2012, and since then, sporadic cases were reported almost every year until the first large outbreak occurred in 2018, followed by the second large outbreak in 2022 after a four-year inter-epidemic period. We were able to generate, for the first time in Rwanda, the genome sequences of RVF viruses that circulated in both outbreaks, and we also identified their genetic relatedness as well as their closest relatives from other countries.

The findings from this study suggested that RVFV was likely introduced to Rwandan territory as a result of the spread of the 2006/2007 East Africa RVF outbreak that concurrently occurred in Kenya, Tanzania, and Somalia. A close phylogenetic relatedness was observed between viruses isolated in Rwanda (2018 and 2022) and those identified in Uganda (2016–2019) whose ancestral origin was linked to the 2006/2007 East African RVF outbreak [22,24]. The Republic of Uganda, which has had an established National Viral Hemorrhagic Fever Surveillance System since 2010, reported RVFV in 2016 for the first time in 48 years, where three human cases of RVFV infection were confirmed during a localized outbreak in Kabale district, which shares borders with Rwanda [24]. Earlier serological studies analyzing livestock samples collected in Uganda in 2009/2010 and in Rwanda in 2012 showed evidence of RVFV circulation in both countries a few years after the end of the above-mentioned East Africa RVF outbreak [56,75]. This large-scale East Africa RVF outbreak occurred during an El Niño event between December 2006 and July 2007 [76]; three different clades belonging to the lineage C were involved, namely Kenya 1, Kenya 2, and Tanzania 1 [44,54]. Different reports published later highlighted the wide distribution of this outbreak virus lineage across several countries, including Sudan, Namibia, Mayotte, Madagascar, and Uganda [14,15,18,22,28,53]. According to Bird et al. (2007), RVFV movement is a continuous process and may involve a large geographic area [43]. The movement of infected animals or mosquitoes has been reported to facilitate RVFV spread. In Madagascar, for example, three repeated RVFV introductions were linked to animal importation from mainland Africa [15], and similar findings were observed in the 2000 Saudi Arabia RVF outbreak, which was associated with small ruminant importation from East Africa [43]. In Rwanda, following the initiation of a national program for cattle distribution among poor families in 2006, named “One Cow Per One Family”, there was subsequently a sharp increase in the importation of improved cattle breeds [77], mostly from East Africa. In addition, the porous nature of Rwandan borders is known to create shortcuts for uncontrolled transboundary movement of livestock [78]. The movement of animals has been documented worldwide as one of the main risk factors for the spread of major transboundary animal diseases [11,79].

Our findings also indicate that a single RVFV lineage C circulated in both the Rwanda 2018 and 2022 outbreaks, and a finer characterization is needed to identify sub-lineages associated with the larger predominant lineage C circulating in the East African region. All Rwanda wild-type RVFV sequences were assigned to RVFV lineage C and exhibited a higher nucleotide identity, suggesting that a resident virus genotype that caused the 2018 outbreak likely re-emerged in the 2022 outbreak. According to available reports, the occurrence of sporadic RVF cases continued in Rwanda after the end of the 2018 RVF epizootic through 2020 [74]. These findings would therefore signal the possible ongoing endemic maintenance of a single RVFV lineage in Rwanda’s ecosystem. This high likelihood of RVFV re-emergence in a previously infected area has also been observed in Tanzania and Kenya [4,31].

Despite Rwanda’s location in a historical endemic zone of RVFV, the evidence from the analyzed data showed a relatively recent endemicity. The reconstructed time-scaled phylogenetic trees indicate that the progenitor of both Rwanda 2018 and 2022 RVF outbreak wild-type viruses existed around 2010–2013, and interestingly, this period coincided with the first livestock abortion storms suspicious of RVFV in Rwanda [55]. The persistence of a single virus lineage in both Rwandan outbreaks would also support this recent incursion of RVFV to Rwandan soil. The low genomic diversity observed in the large group of 2022 outbreak wild-type viruses in Rwanda (0.12–0.44%) was comparable to that observed in 1977/79 Egyptian and 1987 Mauritanian outbreaks (~0.2%) [43], where a single RVFV lineage exploited a new region [46]. This nucleotide distance was far less than that observed in countries with a long history of RVFV endemicity, like Kenya and Zimbabwe, where multiple virus lineages were reported [43,44,54]. It is important to note that contrary to those countries of long endemicity, such as Kenya and Tanzania, where RVF outbreaks have so far been reported in 55% and 39.2% of total districts, respectively, Rwanda registered livestock cases in 100% of the districts during the 2022 RVF outbreak wave (unpublished data). This is particularly concerning, as all parts of the country are likely to be affected in any future RVF outbreak. Rwanda’s eco-climatic conditions, coupled with the recent substantial increase in the proportion of pure exotic and cross-breed dairy cattle, would contribute to facilitating RVFV establishment and expansion in the country. Different studies have reported increased susceptibility of such animals to RVFV [3,4,56]. Taken together, these results indicate a high potential RVF risk in the country and call for an effective and permanent One Health-based RVFV surveillance and control system in order to prevent or reduce the impact of RVFV in Rwanda.

The recovery of vaccine-derived virus genome sequences from slaughterhouse animals was of interest. A proportion of 6/44 (13.6%) sequenced samples collected during the 2022 outbreak showed a closest identity match with RVFV Clone 13, a live-attenuated vaccine strain used to control the outbreak in Rwanda. This observation indicated that recently vaccinated animals were being sent to the slaughterhouses before the expiration of the vaccine withdrawal period. The recovery of vaccine virus sequences in these animals means they had a good amount of RVFV RNA or viable viral particles circulating in their blood [6] and, in this second case, they would constitute a risk of human exposure to this virus strain, which has not been evaluated for humans [80]. Slaughterhouse workers, veterinarians, butchers, and other handlers of raw meat have been reported elsewhere to be at high risk of RVFV infections through contact with infected animal tissues, blood, and other fluids [7,81]. In Rwanda, the vaccination campaign against RVF is carried out annually, between December and February before the long rainy season, and targets all susceptible livestock, namely cattle, goats, and sheep. The vaccine is administered in districts from farm to farm by public veterinarians who may be assisted by private veterinarians [21]. During the RVFV outbreak, a booster dose is administered to all susceptible animals regardless of previous vaccination status. These results showed that farmers may feel the need to sell their animals any time after vaccination, and therefore the public health protection measures should be reinforced, not only during RVF outbreaks but also during any RVF vaccination campaign that involves live-attenuated vaccine usage.

Another concern associated with the use of a live-attenuated vaccine during outbreaks has been virus-RNA segment reassortment between vaccine- and wild-type viruses. This phenomenon, which occurs when two or more virus genotypes coinfect a cell at the same time and exchange their RNA segments during replication, is known to promote virus genetic diversity and could lead to creation of more virulent strains [41]. In our data, there was no evidence of reassortment, as evidenced by the exact congruency of the three segment phylogenetic trees. It seems that the massive use of the Clone 13 RVFV vaccine (lineage E) during the outbreak may not have resulted in the creation of reassortants with the locally circulating lineage C viruses. Although segment reassortment in segmented viruses has been shown both experimentally and in the field [44,45,82], this finding supports earlier observations that reassortment may not be a common occurrence in RVF viruses [24,43,82]. However, this still needs to be closely monitored through genomic surveillance.

Three major limitations need to be considered when interpreting these findings. First, the low number of RVF virus sequences recovered from the 2018 Rwanda RVF outbreak hampered the ability to analyze more precisely the genetic characteristics of the virus that circulated in the outbreak, as well as limiting a more accurate comparison with their counterparts isolated in the 2022 Rwanda RVF outbreak or elsewhere. Secondly, the samples collected from slaughterhouses during the 2022 outbreak, although offering the advantage of an increased chance of sampling the virus during the early to peak stage of viremia, their information on the exact locality of animal origin was missing, and this hindered the ability to depict the exact geographical locations associated with viruses sequenced for 2022 RVF outbreak. Thirdly, the inability to recover the sequences in full length and the exclusion of the S segment short sequences for the vaccine-derived viruses also interfered with our aim of describing these virus strains at the finest resolution.

## 5. Conclusions

Although we could not produce complete RVFV genome sequences for many samples, the generation of a large number of sequences with high genome coverage, the first of its kind for Rwanda, allowed the analysis of the ancestral origin and genetic lineages of circulating RVFV. The results showed that a single RVFV lineage C circulated in both the Rwandan 2018 and 2022 RVF outbreaks and likely arrived in Rwanda as a result of the expansion of the 2006/2007 East Africa RVF outbreak that occurred in Kenya, Tanzania, and Somalia. Our findings provided further evidence on the ongoing widespread circulation of this lineage in Africa. Genetic evidence of the RVFV Clone 13 vaccine strain found in slaughterhouse animals demonstrated a possible occupational risk to people working in meat-related industries. Together, these findings underscore the need for efficient national and international multi-disciplinary collaboration in order to fight and control this emerging transboundary zoonosis.

## Data Availability

RVFV genome sequences generated and used in this study were deposited in GenBank. Details of sequence accessions are provided in the Supplementary Materials under Table S4.

## Supplementary Materials

The following supporting information can be downloaded at: https://www.mdpi.com/article/10.3390/v16071148/s1, Figure S1: Maximum Likelihood (ML) phylogenetic tree based on RVFV L segment; Figure S2: ML phylogenetic tree based on RVFV M segment; Figure S3: ML phylogenetic tree based on RVFV S segment; Tables S1–S3: RVFV L, M and S Segment reference sequences selected from GenBank for phylogenetic analysis, Table S4: Summary of RVFV genome sequences obtained from Rwanda during the study and GenBank accession numbers; Table S5: Summary of 2018 Rift Valley fever outbreak samples screened and sequenced; Table S6: Summary of 2022 Rift Valley fever outbreak positive samples re-screened and sequenced; Table S7: Lineage assignment output using the RVFV M Segment sequences.
