# Supplementary material for: Genomic Epidemiology of Rift Valley Fever Virus Involved in the 2018 and 2022 Outbreaks in Livestock in Rwanda
Source: Viruses. 2024 Jul 17;16(7):1148. doi: 10.3390/v16071148 (PMC11281637; doi:10.3390/v16071148)
Supplement: Supplementary file 1 [file viruses-16-01148-s001.zip › viruses-3084156-supplementary.pdf]

Supplementary materials

Figure S1. Maximum Likelihood phylogenetic tree based on RVFV L segment

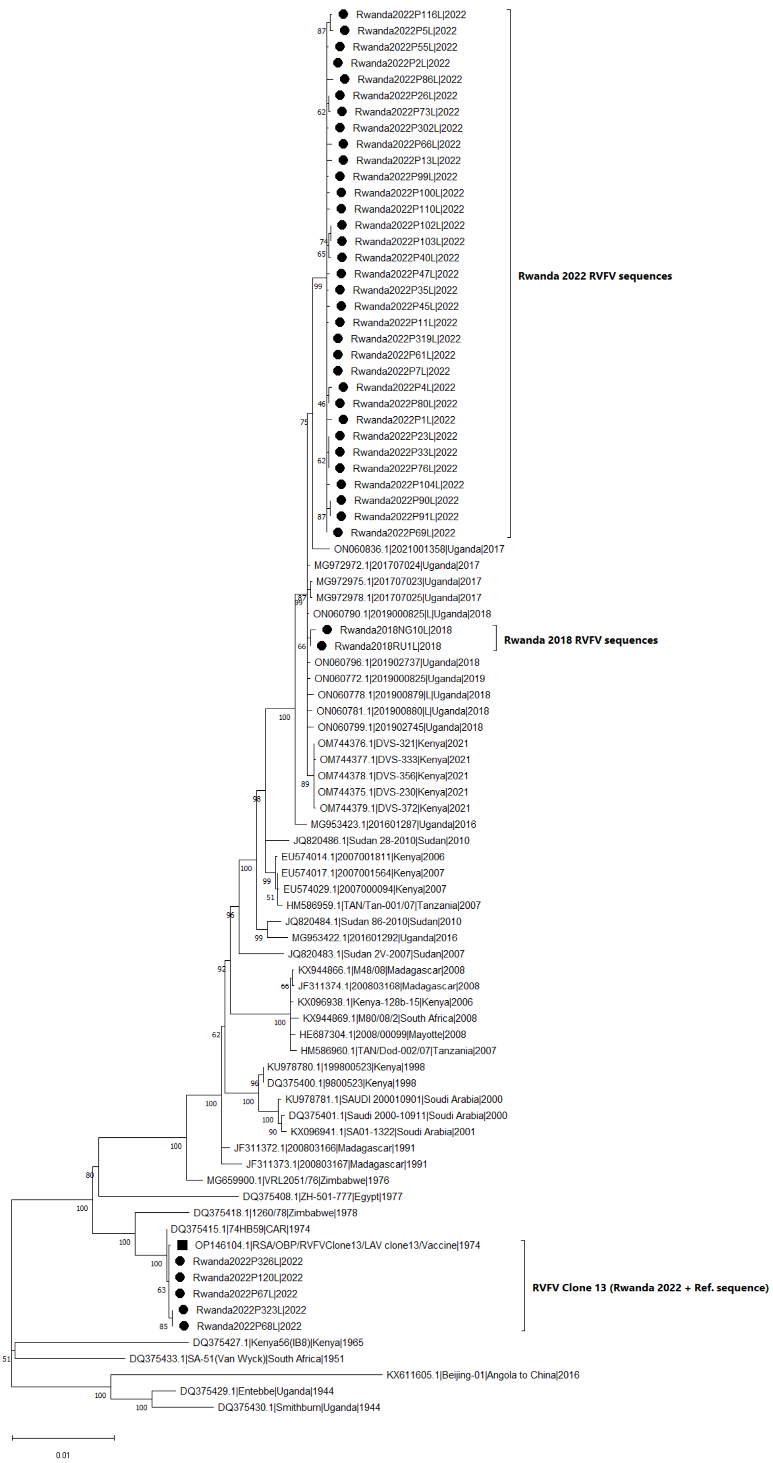

Figure S2. Maximum Likelihood phylogenetic tree based on RVFV M segment

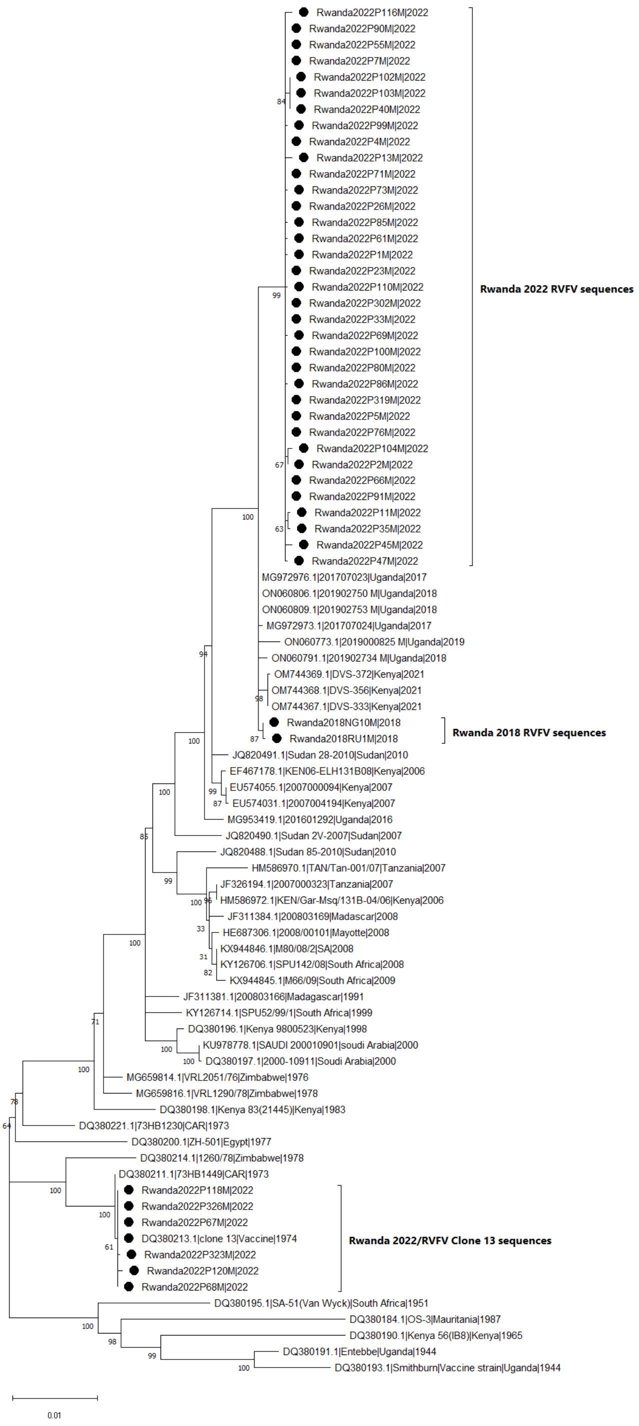

Figure S3. Maximum Likelihood phylogenetic tree based on RVFV S segment

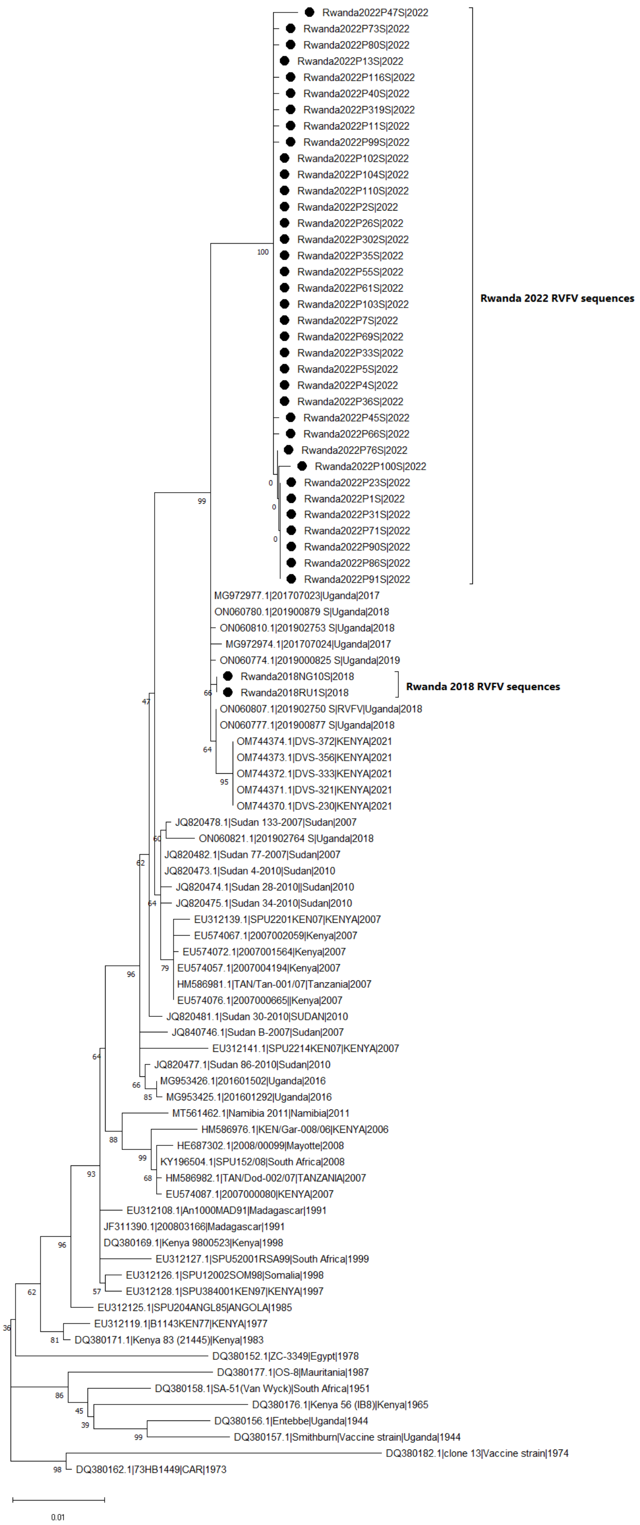

Table S1. RVFV L Segment reference sequences selected from GenBank for phylogenetic analysis

Isolate	No bp	Host species	Year of isolation	Country	Genbank Accession No
Kenya 9800523	6404	Human	1998	Kenya	DQ375400.1
Sudan 2V-2007	6397	Human	2007	Sudan	JQ820483.1
Kenya-128b-15	6404	*Aedes ochraceus*	2006	Kenya	KX096938.1
RL2051/76	6404	Human	1976	Zimbabwe	MG659900.1
M48/08	6404	Bovine	2008	Madagascar	KX944866.1
200803168	6404	Bovine	2008	Madagascar	JF311374.1
Saudi 2000-10911	6404	Human	2000	Soudi Arabia	DQ375401.1
AUDI 200010901	6441	Human	2000	Soudi Arabia	KU978781.1
SA01-1322	6404	*Aedes vexans arabiensis*	2001	Soudi Arabia	KX096941.1
2008/00099	6404	Human	2008	Mayotte	HE687304.1
M80/08/2	6392	Bovine	2008	SA	KX944869.1
TAN/Dod-002/07	6404	Human	2007	Tanzania	HM586960.1
RSA/OBP/RVFVClone13/ LAV clone 13	6404	Derived from RVFV 74HB59	1974	CAR	OP146104.
strain 74HB59	6404	Human	1974	CAR	DQ375415.1
strain 1260/78	6404	Bovine	1978	Zimbabwe	DQ375418.1
strain Entebbe	6404	Mosquito	1944	Uganda	DQ375429.1
Smithburn	6404	Derived from Entebbe strain	1944	Uganda	DQ375430.1
Kenya 56 (IB8)	6404	Bovine	1965	Kenya	DQ375427.1
Beijing-01	6404	Human	2016	China	KX611605.1
ZH-501-777	6404	Plaque pick of ZH 501	1977	Egypt	DQ375408.1
OS-3	6404	Human	1987	Mauritania	DQ375396.
SA-51 (Van Wyck)	6404	Ovine	1951	South Africa	DQ375433.1

Table S2. RVFV M Segment reference sequences selected from GenBank for phylogenetic analysis

Isolate	Length (bp)	Host species	Year of isolation	Country	Accession number
201707023	3879	Human	2017	Uganda	MG972976.1
201902750_M|Uganda|2018	3867	Human	2018	Uganda	ON060806.1
201902753_M|Uganda|2018	3867	Human	2018	Uganda	ON060809.1
201707024	3852	Human	2017	Uganda	MG972973.1
201902734_M|Uganda|2018	3869	Human	2018	Uganda	ON060791.1
DVS-356	3874	Bovine	2021	Kenya	OM744368.1
DVS-333	3879	Bovine	2021	Kenya	OM744367.1
DVS-372	3881	Bovine	2021	Kenya	OM744369.1
2019000825_M|Uganda|2019	3876	Human	2019	Uganda	ON060773.1
2007000094	3885	Bovine	2007	Kenya	EU574055.1
Sudan 85-2010	3885	Human	2010	Sudan	JQ820488.1
KEN06-ELH131B08	3885	*Aedes ochraceus*	2006	Kenya	EF467178.1
Sudan 28-2010	3884	Human	2010	Sudan	JQ820491.1
2007004194	3885	Bovine	2007	Kenya	EU574031.1
201601292	3849	Human	2016	Uganda	MG953419.1
Sudan 2V-2007	3871	Human	2007	Sudan	JQ820490.1
200803166	3885	Human	1991	Madagascar	JF311381.1
SPU52/99/1	3885	Buffalo	1991	South Africa	KY126714.1
Kenya 9800523	3885	Human	1998	Kenya	DQ380196.1
M80/08/2	3879	Bovine	2008	South Africa	KX944846.1
SAUDI 200010901	3952	Human	2000	Soudi Arabia	KU978778.1
VRL2051/76	3885	Human	1976	Zimbabwe	MG659814.1
SPU142/08	3885	Bovine	2008	South Africa	KY126706.1
2008/00101	3885	Bovine	2008	Mayotte	HE687306.1
2007000323	3885	Human	2007	Tanzania	JF326194.1
200803169	3885	Bovine	2008	Madagascar	JF311384.1
Saudi 2000-10911	3885	Human	2000	Soudi Arabia	DQ380197.1
KEN/Gar-Msq/131B-04/06	3885	Bovine	2006	Kenya	HM586972.1
Sudan 85-2010	3885	Human	2010	Sudan	JQ820488.1
M66/09	3877	Bovine	2009	South Africa	KX944845.1
VRL1290/78	3885	Bovine	1978	Zimbabwe	MG659816.1
TAN/Tan-001/07	3885	Bovine	2007	Tanzania	HM586970.1
Kenya 83 (21445)	3885	*Aedes*	1983	Kenya	DQ380198.1

		mucintochi			
clone 13	3885	Derived from 74HB59	N/A	N/A	DQ380213.1
strain 73HB1449	3885	Human	1973	CAR	DQ380211.1
strain 1260/78	3885	Bovine	1978	Zimbabwe	DQ380214.1
strain 73HB1230	3885	Human	1973	CAR	DQ380221.1
Entebbe strain	3885	Mosquito	1944	Uganda	DQ380191.1
Smithburn	3885	Derived from Entebbe strain	1944	Uganda	DQ380193.1
Kenya 56 (IB8)	3885	Bovine	Pre-1965	Kenya	DQ380190.1
Beijing-01	3885	Human	2016	Angola-China	KX611606.1
ZH-501	3885	Human	1977	Egypt	DQ380200.1
OS-3	3885	Human	1987	Mauritania	DQ380184.1
SA-51	3885	Ovine	1951	South Africa	DQ380195.1

Table S3. RVFV S segment reference sequences selected from GenBank for phylogenetic analysis

**Isolate**	**Length bp**	**Host species**	**Year of isolation**	**Country**	**Accession No**
201707023	1686	Human	2017	Uganda	MG972977.1
201902753_S	1680	human	2018	Uganda	ON060810.1
201902750_S	1679	Human	2018	uganda	ON060807.1
201900879_S	1680	Human	2018	Uganda	ON060780.1
201900877_S| Uganda|2018	1678	Human	2018	Uganda	ON060777.1
2019000825_S| Uganda|2019	1683	Human	2019	Uganda	ON060774.1
201707024	1644	Human	2017	Uganda	MG972974.1
Sudan 77-2007	1689	Human	2007	Sudan	JQ820482.1
Sudan 133-2007	1690	Human	2007	Sudan	JQ820478.1
Sudan 86-2010	1691	Human	2010	Sudan	JQ820477.1
Sudan 4-2010	1690	Human	2010	Sudan	JQ820473.1
TAN/Tan-001/07	1691	Not available	2007	Tanzania	HM586981.1
2007000665	1690	Bovine	2007	Kenya	EU574076.1
2007001564	1690	Bovine	2007	Kenya	EU574072.1
2007004194	1689	Bovine	2007	Kenya	EU574057.1
Sudan B-2007	1690	Human	2007	Sudan	JQ840746.1
Sudan 30-2010	1689	Human	2010	Sudan	JQ820481.1
Sudan 28-2010	1690	Human	2010	Sudan	JQ820474.1
Sudan 34-2010	1690	Human	2010	Sudan	JQ820475.1
2007002059	1690	Ovine	2007	Kenya	EU574067.1

200803166	1691	Human	1991	Madagascar	JF311390.1
SPU2201KEN07	1690	Human	2007	Kenya	EU312139.1
Kenya 9800523	1690	Human	1998	Kenya	DQ380169.1
201601502	1641	Human	2016	Uganda	MG953426.1
201902764_S|Uganda|2018	1678	Human	2018	Uganda	ON060821.1
An1000MAD91	1691	Bovine	1991	Madagascar	EU312108.1
SPU12002SOM98	1690	Caprine	1998	Somalia	EU312126.1
201601292	1675	Human	2016	Uganda	MG953425.1
Namibia_2011	1690	springbok	2011	Namibia	MT561462.1
KEN/Gar-008/06	1691	Not available	2006	Kenya	HM586976.1
SPU2214KEN07	1691	Human	2007	Kenya	EU312141.1
SPU384001KEN97	1690	Human	1997	Kenya	EU312128.1
B1143KEN77	1691	Not available	1977	Kenya	EU312119.1
DVS-372	1690	Bovine	2021	Kenya	OM744374.1
DVS-356	1689	Bovine	2021	Kenya	OM744373.1
DVS-333	1689	Bovine	2021	Kenya	OM744372.1
DVS-321	1690	Bovine	2021	Kenya	OM744371.1
DVS-230	1689	Bovine	2021	Kenya	OM744370.1
SPU152/08	1692	Human	2008	South Africa	KY196504.1
2008/00099	1691	Human	2008	Mayotte	HE687302.1
TAN/Dod-002/07	1691	not given	2007	Tanzania	HM586982.1
SPU52001RSA99	1691	Buffalo	1999	South Africa	EU312127.1
SPU204ANGL85	1691	Human	1985	Angola	EU312125.1
2007000080	1691	Bovine	2007	Kenya	EU574087.1
Kenya 83 (21445)	1691	*Aedes mucintoshi*	1983	Kenya	DQ380171.1
clone 13	1141	Plaque pick of RVFV 74HB59	1974	CAR	DQ380182.1
73HB1449	1690	Human	1973	CAR	DQ380162.1
Entebbe strain	1691	Mosquito	1944	Uganda	DQ380156.1
Smithburn	1691	Derived from Entebbe strain	1944	Uganda	DQ380157.1
Kenya 56 (IB8)	1690	Bovine	before 1965	Kenya	DQ380176.1
BJ01	1686	Human	2016	Angola to China	KX632068.1
ZC-3349	1690	Bovine	1978	Egypt	DQ380152.1
OS-8	1690	Human	1987	Mauritania	DQ380177.1
SA-51 (Van Wyck)	1691	Ovine	1951	South Africa	DQ380158.1

Table S4. Summary of RVFV genome sequences obtained from Rwanda during the study, and Genbank accession numbers

Isolate name	Date of collection	Abattoir (district)	Species/Specimen type	Accession numbers		
				L segment	M segment	S segment
Rwanda 2018NG10	7 Jun 2018	Ngoma	Bovine/Blood	PP747231	PP747236	PP747241
Rwanda 2018RU1	9 Jul 2018	Rulindo	Bovine/Blood	PP747233	PP747238	PP747243
Rwanda 2022P1	14 Jun 2022	Nyarugenge	Bovine/plasma	PP746304	PP746342	PP746379
Rwanda 2022P2	7 Jun 2022	Nyarugenge	Bovine/plasma	PP746314	PP746351	PP746388
Rwanda 2022P4	7 Jun 2022	Nyarugenge	Bovine/plasma	PP746323	PP746359	PP746397
Rwanda 2022P5	3 Jun 2022	Nyarugenge	Bovine/plasma	PP746327	PP746363	PP746401
Rwanda 2022P7	9 Jun 2022	Kamonyi	Bovine/plasma	PP746334	PP746369	PP746406
Rwanda 2022P11	9 Jun 2022	Kamonyi	Bovine/plasma	PP746309	PP746347	PP746384
Rwanda 2022P13	8 Jun 2022	Kamonyi	Bovine/plasma	PP746313	PP746350	PP746387
Rwanda 2022P23	13 Jun 2022	Muhanga	Bovine/plasma	PP746315	PP746352	PP746389
Rwanda 2022P26	11Jun 2022	Muhanga	Bovine/plasma	PP746316	PP746353	PP746390
Rwanda 2022P31	17 Jun 2022	Nyarugenge	Bovine/plasma	-	-	PP746392
Rwanda 2022P33	25 Jun 2022	Nyarugenge	Bovine/plasma	PP746321	PP746357	PP746394
Rwanda 2022P35	17 Jun 2022	Nyarugenge	Bovine/plasma	PP746322	PP746358	PP746395
Rwanda 2022P36	25 Jun 2022	Nyarugenge	Bovine/plasma	-	-	PP746396
Rwanda 2022P40	3 Jul 2022	Muhanga	Bovine/plasma	PP746324	PP746360	PP746398
Rwanda 2022P45	17 Jul 2022	Muhanga	Bovine/plasma	PP746325	PP746361	PP746399
Rwanda 2022P47	9 Jun 2022	Kamonyi	Bovine/plasma	PP746326	PP746362	PP746400
Rwanda 2022P55	12 Jun 2022	Gicumbi	Bovine/plasma	PP746328	PP746364	PP746402
Rwanda 2022P61	22 Jun 2022	Kamonyi	Bovine/plasma	PP746329	PP746365	PP746403
Rwanda 2022P66	22 Jun 2022	Nyarugenge	Bovine/plasma	PP746330	PP746366	PP746404
Rwanda 2022P67*	18 Jun 2022	Muhanga	Bovine/plasma	PP746331	PP746367	-
Rwanda 2022P68*	18 Jun 2022	Muhanga	Bovine/plasma	PP746332	-	-
Rwanda 2022P69	18 Jun 2022	Gicumbi	Bovine/plasma	PP746333	PP746368	PP746405
Rwanda 2022P71	14 Jun 2022	Nyarugenge	Bovine/plasma	-	PP746370	PP746407
Rwanda 2022P73	3 Jun 2022	Muhanga	Ovine/plasma	PP746335	PP746371	PP746408
Rwanda 2022P76	27Jun 2022	Muhanga	Bovine/plasma	PP746336	PP746372	PP746409
Rwanda 2022P80	28 Jun 2022	Nyarugenge	Bovine/plasma	PP746337	PP746373	PP746410
Rwanda 2022P85	28 Jun 2022	Nyarugenge	Bovine/plasma	-	PP746374	-
Rwanda 2022P86	28 Jun 2022	Nyarugenge	Bovine/plasma	PP746338	PP746375	PP746411
Rwanda 2022P90	9 July 2022	Nyarugenge	Bovine/plasma	PP746339	PP746376	PP746412
Rwanda 2022P91	9 July 2022	Nyarugenge	Bovine/plasma	PP746340	PP746377	PP746413
Rwanda 2022P99	29 Jul 2022	Nyarugenge	Bovine/plasma	PP746414	PP746378	PP746341
Rwanda 2022P100	31 Jul 2022	Gicumbi	Bovine/plasma	PP746305	PP746343	PP746380
Rwanda 2022P102	5 Aug 2022	Gicumbi	Bovine/plasma	PP746306	PP746344	PP746381
Rwanda 2022P103	8 Aug 2022	Gicumbi	Bovine/plasma	PP746307	PP746345	PP746382
Rwanda 2022P104	8 Aug 2022	Gicumbi	Bovine/plasma	PP746308	PP746346	PP746383
Rwanda 2022P110	12 Aug 2022	Nyaruguru	Bovine/plasma	PP746310	PP746348	PP746385
Rwanda 2022P116	1 Jul 2022	Nyanza	Bovine/plasma	PP746311	PP746349	PP746386

Rwanda 2022P118*	8 Jul 2022	Huye	Ovine/plasma	-	-	-
Rwanda 2022P120*	8 Jul 2022	Gisagara	Ovine/plasma	PP746312	-	-
Rwanda 2022P302	4 Jun 2022	Rusizi	Bovine/plasma	PP746317	PP746354	PP746391
Rwanda 2022P319	25 Aug 2022	Nyamasheke	Bovine/plasma	PP746318	PP746355	PP746393
Rwanda 2022P323*	2 Sep 2022	Kamonyi	Bovine/plasma	PP746319	-	-
Rwanda 2022P326*	28 Sep 2022	Nyarugenge	Bovine/plasma	PP746320	PP746356	-

A dash (-): no sequence data with at least 90% genome coverage was obtained. A star (*): RVFV clone 13 sequence

Table S5. Summary of 2018 Rift Valley fever outbreak samples screened and sequenced.

District	Animal species	Sample type	Total	No + /RT-qPCR*	No sequenced	No sequences
Eastern Province						
Kayonza	Bovine	Blood	8	3	1	0
Rwamagana	Bovine	Blood	5	0	0	0
Gatsibo	Caprine	Blood	4	1	0	0
Ngoma	Bovine	Blood	2	1	1	1
	Bovine	Serum	1	0	0	0
Kirehe	Bovine	Blood	1	0	0	0
Nyagatare	Bovine	Blood	3	1	0	0
	Bovine	Serum	4	0	0	0
Northern Province						
Rulindo	Bovine	Blood	5	3	1	1
	Bovine	Blood	4	2	1	0
Total			37	11	4	2

RT-qPCR: Reverse Transcription Real-Time Polymerase Chain Reaction

Table S6. Summary of 2022 Rift Valley fever outbreak positive samples re-screened and sequenced.

**Slaughter site (district)**	**Bovine**	**Caprine**	**Ovine**	**Total samples**	**Positive/pre-sequencing RT-qPCR***	**N° sequenced**	**N° sequences/ segment**		
							**L**	**M**	**S**
Kigali-Rubirizi testing center									
Nyarugenge	36	4	3	43	33	18	13	15	15
Kicukiro	10	0	0	10	5	0	0	0	0
Bugesera	0	1	0	1	0	0	0	0	0
Gicumbi	11	0	0	11	9	6	6	6	6
Kamonyi	16	1	0	17	14	6	6	6	5

Muhanga	23	0	3	26	17	8	8	8	6
Ruhango	3	0	0	3	2	0	0	0	0
Huye testing center									
Huye	0	1	4	5	4	1	0	1	0
Gisagara	1	3	2	6	2	1	1	1	0
Nyamagabe	9	0	0	9	4	0	0	0	0
Nyanza	1	0	0	1	1	1	1	1	1
Nyaruguru	2	2	0	4	1	1	1	1	1
Nyamasheke testing center									
Nyamasheke	6	4	0	10	6	1	1	1	1
Rusizi	9	2	0	11	4	1	1	1	1
**Total**	**127**	**18**	**12**	**157**	**102**	**44**	**38**	**41**	**36**

RT-qPCR: Reverse Transcription Real-Time Polymerase Chain Reaction

Table S7. Lineage assignment output of the online RVFV typing tool (version 0.2) using RVFV M Segment sequences

**Name**	**Length (bp)**	**Begin**	**End**	**Type**	**Type support**
Rwanda2018NG10M|2018	3840	25	3864	Lineage C	99.0
Rwanda2018RU1M|2018	3840	25	3864	Lineage C	99.0
Rwanda2022P1M|2022	3513	352	3864	Lineage C	99.0
Rwanda2022P100M|2022	3561	304	3864	Lineage C	100.0
Rwanda2022P102M|2022	3534	331	3864	Lineage C	98.0
Rwanda2022P103M|2022	3541	324	3864	Lineage C	99.0
Rwanda2022P104M|2022	3570	295	3864	Lineage C	99.0
Rwanda2022P11M|2022	3606	259	3864	Lineage C	99.0
Rwanda2022P110M|2022	3567	298	3864	Lineage C	100.0
Rwanda2022P116M|2022	3582	283	3864	Lineage C	100.0
Rwanda2022P118M|2022	3442	352	3864	Lineage E	89.0
Rwanda2022P120M|2022	3368	344	3864	Lineage E	87.0
Rwanda2022P13M|2022	3558	307	3864	Lineage C	98.0
Rwanda2022P2M|2022	3517	348	3864	Lineage C	98.0
Rwanda2022P23M|2022	3847	25	3871	Lineage C	100.0
Rwanda2022P26M|2022	3560	305	3864	Lineage C	100.0
Rwanda2022P302M|2022	3541	324	3864	Lineage C	99.0
Rwanda2022P319M|2022	3661	83	3864	Lineage C	98.0
Rwanda2022P323M|2022	3307	352	3864	Lineage E	92.0
Rwanda2022P326M|2022	3513	352	3864	Lineage E	90.0
Rwanda2022P33M|2022	3513	352	3864	Lineage C	100.0
Rwanda2022P35M|2022	3841	25	3864	Lineage C	98.0
Rwanda2022P4M|2022	3513	352	3864	Lineage C	99.0
Rwanda2022P40M|2022	3558	307	3864	Lineage C	99.0

Rwanda2022P45M|2022	3731	134	3864	Lineage C	100.0
Rwanda2022P47M|2022	3840	25	3864	Lineage C	99.0
Rwanda2022P5M|2022	3513	352	3864	Lineage C	99.0
Rwanda2022P55M|2022	3568	297	3864	Lineage C	99.0
Rwanda2022P61M|2022	3571	294	3864	Lineage C	100.0
Rwanda2022P66M|2022	3840	25	3864	Lineage C	100.0
Rwanda2022P67M|2022	3596	269	3864	Lineage E	84.0
Rwanda2022P68M|2022	3303	304	3864	Lineage E	86.0
Rwanda2022P69M|2022	3513	352	3864	Lineage C	99.0
Rwanda2022P7M|2022	3529	336	3864	Lineage C	99.0
Rwanda2022P71M|2022	3513	352	3864	Lineage C	100.0
Rwanda2022P73M|2022	3561	304	3864	Lineage C	98.0
Rwanda2022P76M|2022	3858	25	3882	Lineage C	100.0
Rwanda2022P80M|2022	3710	36	3864	Lineage C	100.0
Rwanda2022P85M|2022	3513	352	3864	Lineage C	99.0
Rwanda2022P86M|2022	3619	225	3864	Lineage C	97.0
Rwanda2022P90M|2022	3531	334	3864	Lineage C	99.0
Rwanda2022P91M|2022	3513	352	3864	Lineage C	97.0
Rwanda2022P99M|2022	3513	352	3864	Lineage C	99.0
MG972976.1|201707023|Uganda	3879	4	3882	Lineage C	100.0
ON060806.1|201902750_M|Uganda	3867	11	3877	Lineage C	100.0
ON060809.1|201902753_M|Uganda	3867	11	3877	Lineage C	100.0
MG972973.1|201707024|Uganda	3852	16	3867	Lineage C	98.0
ON060791.1|201902734_M|Uganda	3869	11	3878	Lineage C	96.0
OM744368.1|DVS-356|Kenya|2021	3874	4	3877	Lineage C	99.0
OM744367.1|DVS-333|Kenya|2021	3879	4	3882	Lineage C	100.0
OM744369.1|DVS-372|Kenya|2021	3881	1	3881	Lineage C	99.0
ON060773.1|2019000825_M|Uganda	3876	3	3878	Lineage C	99.0
EU574055.1|2007000094|Kenya	3885	1	3885	Lineage C	88.0
JQ820488.1|Sudan_85-2010|Sudan	3885	1	3885	Lineage C	75.0
EF467178.1|KEN06-ELH131B08|Ken.	3885	1	3885	Lineage C	89.0
JQ820491.1|Sudan_28-2010|Sudan	3884	1	3884	Lineage C	76.0
EU574031.1|2007004194|Kenya	3885	1	3885	Lineage C	89.0
MG953419.1|201601292|Uganda	3841	20	3860	Lineage C	94.0
JQ820490.1|Sudan_2V-2007|Sudan	3871	12	3882	Lineage C	87.0
JF311381.1|200803166|Madagasca	3885	1	3885	Lineage C	75.0
KY126714.1|SPU52991|South_Africa	3885	1	3885	Lineage C	93.0
DQ380196.1|Kenya_9800523|Kenya	3885	1	3885	Lineage C	79.0
KX944846.1|M80082|SA|2008	3879	7	3885	Lineage C	78.0
KU978778.1|SAUDI_200010901|SA	3952	1	3885	Lineage C	88.0
MG659814.1|VRL205176|Zimbabwe	3885	1	3885	Lineage C	80.0
KY126706.1|SPU14208|South_Africa	3885	1	3885	Lineage C	78.0
HE687306.1|200800101|Mayotte	3885	1	3885	Lineage C	82.0
JF326194.1|2007000323|Tanzania	3885	1	3885	Lineage C	76.0
JF311384.1|200803169|Madascar	3885	1	3885	Lineage C	84.0
DQ380197.1|2000-10911|Soudi_A.	3885	1	3885	Lineage C	81.0

HM586972.1|KENGar-Msq131B-0406	3885	1	3885	Lineage C	83.0
KX944845.1|M6609|South_Africa	3877	9	3885	Lineage C	81.0
MG659816.1|VRL129078|Zimbabwe	3885	1	3885	Lineage C	84.0
HM586970.1|TANTan-00107|Tanz.	3815	71	3885	Lineage C	79.0
DQ380198.1|Kenya_8321445|Kenya	3885	1	3885	Lineage C	99.0
DQ380213.1|clone_13|Vaccine	3885	1	3885	Lineage E	86.0
DQ380211.1|73HB1449|CAR|1973	3885	1	3885	Lineage E	87.0
DQ380214.1|126078|Zimbabwe|1978	3885	1	3885	Lineage E	87.0
DQ380221.1|73HB1230|CAR|1973	3885	1	3885	Lineage D	82.0
DQ380191.1|Entebbe|Uganda|1944	3885	1	3885	Lineage K	95.0
DQ380193.1|Smithburn|Vaccine	3885	1	3885	Lineage K	97.0
DQ380190.1|Kenya_56IB8|Kenya	3885	1	3885	Lineage L	100.0
DQ380200.1|ZH-501|Egypt|1977	3885	1	3885	Lineage A	89.0
DQ380184.1|OS-3|Mauritania|1988	3885	1	3885	Lineage N	100.0
DQ380195.1|SA-51Van_Wyck|S.A	3885	1	3885	Lineage O	97.0
